# Tocilizumab may slow radiographic progression in patients with systemic or polyarticular-course juvenile idiopathic arthritis: post hoc radiographic analysis from two randomized controlled trials

**DOI:** 10.1186/s13075-020-02303-y

**Published:** 2020-09-10

**Authors:** Clara Malattia, Nicolino Ruperto, Silvia Pederzoli, Elena Palmisani, Angela Pistorio, Carine Wouters, Pavla Dolezalova, Berit Flato, Stella Garay, Gabriella Giancane, Chris Wells, Wendy Douglass, Hermine I. Brunner, Fabrizio De Benedetti, Angelo Ravelli, for the Paediatric Rheumatology International Trials Organisation (PRINTO) and the Pediatric Rheumatology Collaborative Study Group (PRCSG)

**Affiliations:** 1 Università degli Studi di Genova, Genoa, Italy; 2 IRCCS Istituto Giannina Gaslini, Clinica Pediatrica e Reumatologia, PRINTO, Via G. Gaslini 5, 16147 Genoa, Italy; 3 IRCCS Istituto Giannina Gaslini, Servizio di Epidemiologia e Biostatistica, Genoa, Italy; 4 University Hospital Gasthuisberg, Leuven, Belgium; 5 General University Hospital and First Faculty of Medicine, Charles University, Prague, Czech Republic; 6 Oslo University Hospital and University of Oslo, Oslo, Norway; 7 Hospital Sor Maria Ludovica, La Plata, Argentina; 8 Roche Products Ltd., Welwyn Garden City, UK; 9 Cincinnati Children’s Hospital Medical Center, Cincinnati, OH USA; 10 IRCCS Ospedale Pediatrico Bambino Gesú, Rome, Italy; 11 Sechenov First Moscow State Medical University, Moscow, Russian Federation

**Keywords:** Biologicals, Disease-modifying antirheumatic drugs (DMARDs), Tocilizumab, Systemic juvenile idiopathic arthritis, Polyarticular-course juvenile idiopathic arthritis

## Abstract

**Background:**

Few clinical trials have investigated the prevention of radiographic progression in children with juvenile idiopathic arthritis treated with antirheumatic drugs. This study aimed to investigate radiographic progression in patients with systemic juvenile idiopathic arthritis (sJIA) and patients with polyarticular-course juvenile idiopathic arthritis (pcJIA) treated with the anti–interleukin-6 receptor antibody tocilizumab for 2 years in the TENDER and CHERISH randomized controlled trials, respectively.

**Methods:**

Standard radiographs of both wrists and both hands in the posteroanterior view were obtained within 4 weeks of baseline and were repeated at weeks 52 ± 4 and 104 ± 4 in both trials. All films were scored by two independent readers using the adapted Sharp–van der Heijde (aSH) and Poznanski scoring methods. Although the Poznanski score indicates bone growth limitation or cartilage growth decrease, which are not the same as joint space narrowing in rheumatoid arthritis, its change reflects damage to cartilage. Therefore, impairment in the Poznanski score as well as the aSH score was considered as a measure of structural joint damage. Radiographic progression was defined as worsening of radiographic scores beyond the smallest detectable difference.

**Results:**

Poznanski and aSH scores were available at baseline and at one or more postbaseline time points for 33 and 47 of 112 sJIA patients and 61 and 87 of 188 pcJIA patients, respectively, providing a representative subset of the study populations. The inter-reader and intra-reader agreement intra-class correlation coefficient was > 0.8. Median baseline Poznanski and aSH scores, respectively, were − 2.4 and 24.6 for sJIA patients and − 1.5 and 8.0 for pcJIA patients. Compared with baseline, aSH scores remained stable for all sJIA patients at week 52, whereas 9.4% of sJIA patients had radiographic progression according to Poznanski scores at week 52; at 104 weeks, radiographic progression according to aSH and Poznanski scores was observed in 5.4% and 11.5%, respectively. In pcJIA patients, radiographic progression from baseline at 52 weeks and at 104 weeks was 12.5% and 2.9%, respectively, using aSH scoring and 6.5% and 4%, respectively, using Poznanski scoring.

**Conclusion:**

Tocilizumab may delay radiographic progression in children with sJIA and children with pcJIA.

**Trial registration:**

Trial registration numbers and dates: TENDER, NCT00642460 (March 19, 2008); CHERISH, NCT00988221 (October 1, 2009)

## Background

Juvenile idiopathic arthritis (JIA) encompasses a heterogeneous group of immune-mediated, chronic, noninfectious joint diseases commencing before patients are 16 years of age [1]. All JIA types are characterized by chronic synovial inflammation, potentially leading to permanent damage of articular cartilage and bone [2]. Joint space narrowing, reflecting cartilage loss over the joint surface, can result in serious impairment of physical function [3]. Many children with JIA develop marked radiographic joint damage, with cartilage loss and erosions often developing early in the course of their illness [4]. Despite the recent development and validation of scoring methods [4–12], the capacity of antirheumatic medications to prevent radiographic progression has not been extensively investigated [13–16].

Tocilizumab is a humanized anti–human interleukin-6 (IL-6) receptor-alpha antibody that inhibits IL-6 signaling [17, 18]. Based on results from phase 3 randomized controlled trials in patients with systemic JIA (sJIA [TENDER trial]) and polyarticular-course JIA (pcJIA [CHERISH trial]) [19, 20], intravenous tocilizumab, in combination with methotrexate or as monotherapy, was approved for the treatment of both subtypes of JIA [21]. Tocilizumab has been shown to significantly slow radiographic joint damage in adults with rheumatoid arthritis [22–24] and to reduce radiographic abnormalities in a small sample of patients with sJIA [25].

The present analysis was conducted to investigate the effect of tocilizumab on radiographic progression for up to 2 years in patients with sJIA or pcJIA from the TENDER and CHERISH phase 3 randomized controlled trials.

## Patients and methods

### Patients

Eligibility criteria have been published [19, 20]. Briefly, eligible patients were 2 to 17 years of age, had a diagnosis of sJIA or pcJIA (rheumatoid factor–positive polyarthritis, rheumatoid factor–negative polyarthritis, or extended oligoarthritis) according to the International League of Associations for Rheumatology classification criteria [1], and had active disease for ≥ 6 months. At enrollment, patients with sJIA had to have ≥ 5 active joints or fever > 38 °C and ≥ 2 active joints and inadequate response to nonsteroidal anti-inflammatory drugs and glucocorticoids. Patients with pcJIA had to have ≥ 5 active joints and inadequate response or intolerance to methotrexate. Stable doses of methotrexate, nonsteroidal anti-inflammatory drugs, and/or oral glucocorticoids (maximum dose 0.5 mg/kg/day for sJIA, 0.2 mg/kg/day for pcJIA) were allowed throughout the studies. Children with wrist involvement were included if consent for X-ray was provided.

### Study design

The sJIA trial was conducted across 43 centers in 17 countries, and the pcJIA trial was conducted at 58 centers in 15 countries. All centers are part of the Paediatric Rheumatology International Trials Organisation (PRINTO) [26] or the Pediatric Rheumatology Collaborative Study Group (PRCSG). Study designs have been described [19, 20]. Briefly, the sJIA trial was a 5-year, phase 3 study of the efficacy and safety of tocilizumab in patients with active sJIA, with a 12-week, randomized, double-blind, placebo-controlled period followed by a long-term extension. The pcJIA trial was a 2-year, phase 3 randomized withdrawal trial of the efficacy and safety of tocilizumab in patients with pcJIA, with a 16-week open-label, lead-in period; a 24-week double-blind, placebo-controlled withdrawal period; and a long-term extension. Both trials were conducted in accordance with the Declaration of Helsinki and good clinical practice and were approved by the local institutional review board or the independent ethics committees at each center.

### Radiographic assessments

Standard radiographs of both wrists and both hands in the posteroanterior view were obtained for a subset of patients who consented to X-ray within 4 weeks of baseline and again at week 52 (± 4 weeks) and at week 104 (± 4 weeks). All radiographs were scored independently by two pediatric rheumatologists (CM, AR) with > 10 and > 20 years of experience, respectively, in clinical and radiographic assessment of children with JIA using the adapted Sharp–van der Heijde (aSH) [11] and Poznanski [27] scoring methods. Radiographs from each patient were read in random order; previous radiographs and scores were not available to readers when they were examining and scoring follow-up radiographs. Inter-reader reliability methods are described in the Additional File.

Calculation of aSH scores was based on assessment of 15 areas for joint space narrowing (JSN) and 21 areas for erosion in each hand and wrist [11]. For each area, JSN was scored from 0 to 4 (0 = normal, 1 = focal or minimal narrowing, 2 = loss of joint space < 50%, 3 = loss of joint space > 50%, 4 = ankylosis) and erosion from 0 to 5 (0 = normal shape, 1 = slight deformity, 2 = moderate deformity, 3 = marked deformity, 4 = severe deformity 5 = extensive bone destruction). aSH scores from each of the two independent readers were averaged for each of the areas assessed. Scores were calculated from the unweighted summary score of 15 area JSN (range, 0–120) and 21 area erosion (range, 0–210), with the total aSH score (range, 0–330) representing the sum of the JSN and erosion scores and higher scores indicating greater damage. Further details are described in the Additional File.

The Poznanski method is based on measurements of the radial metacarpal (RM) width, which is the distance from the base of the third metacarpal bone to the midpoint of the distal growth plate of the radius, and the maximum length of the second metacarpal bone (M2). Although the Poznanski score indicates bone growth limitation or cartilage growth decrease, which are not the same as joint space narrowing in rheumatoid arthritis, its change reflects damage to cartilage. Therefore, impairment in the Poznanski score as well as the aSH score was considered as a measure of structural joint damage. For each wrist, the number of standard deviations between the expected and the observed RM width for the measured M2 length was calculated according to the formulae of Poznanski et al. [27]. The RM/M2 ratio, which represents the Poznanski score, reflects the amount of radiographic damage in the wrist. The more negative the Poznanski score, the more severe the radiographic damage. Further details are described in the Additional File.

Importantly, the radiographic scores obtained with the two methods used in our study go in opposite directions. With the Poznanski score, a decrease is abnormal, whereas with the aSH score an increase is abnormal.

Key radiographic end points were change from baseline in radiographic scores, proportion of patients without radiographic progression from baseline to weeks 52 or 104, and relationship between radiographic progression and clinical response.

Radiographic progression at 1 and 2 years was determined by subtracting baseline scores from week 52 and 104 scores. Radiographic progression for aSH and Poznanski scores was defined using the smallest detectable difference (SDD) and the zero value (Additional File) [28]. Positive change in aSH score [11] or negative change in Poznanski score [27], or both, was considered indicative of radiographic progression. Data are reported using Bland and Altman plots. Further details are described in the Additional File.

Change from baseline to week 104 in aSH score was analyzed for patients in the pcJIA radiographic population by stratifying patients according to baseline methotrexate use (yes/no), baseline glucocorticoid use (yes/no), previous biologic use (yes/no), disease duration (< 2 years/≥ 2 years), and rheumatoid factor (positive/negative/missing).

### Statistical analysis

Radiographic end points were evaluated for the entire radiographic population of the sJIA trial, whereas analysis of the pcJIA trial focused on the subgroup of radiographic patients who received tocilizumab continuously throughout the study (those randomly assigned to receive tocilizumab during the double-blind period [continuous TCZ population]). Some radiographic analyses were performed for the entire radiographic population (all patients with radiographic data regardless of randomly assigned placebo or tocilizumab treatment [all TCZ population]). The aSH population included all patients who received a dose of tocilizumab and had a baseline and ≥ 1 postbaseline (week 52 or week 104) score, with data at weeks 52 and 104 summarized for patients who received tocilizumab continuously. The Poznanski population included all patients who received ≥ 1 dose of tocilizumab and had ≥ 1 postbaseline (week 52 or week 104) score, with data at weeks 52 and 104 summarized for patients who received tocilizumab continuously.

Missing readings of areas for JSN or erosion in the aSH score, because of either absence of a particular area due to incomplete ossification (typically seen in younger children) or technical inadequacy of the X-ray film, were imputed using average readings from other readable areas. If readings from an entire hand or wrist were missing, erosion, JSN, and total adapted aSH scores were set to missing. No imputation was used for missing Poznanski score data or other analyses.

Changes from baseline in aSH and Poznanski scores were analyzed using the Wilcoxon signed rank test. Correlation between aSH and Posnanski scores was assessed using Pearson and Spearman correlations. Annualized rates of progression were calculated as the change from baseline of each follow-up visit, divided by the number of days between the two assessments, and multiplied by 365.25.

## Results

### Radiographic populations and baseline characteristics

In total, 112 patients were enrolled in the sJIA trial and received ≥ 1 dose of tocilizumab. Baseline and ≥ 1 postbaseline aSH scores and Poznanski scores were assessed for 47 and 33 patients, respectively, from 25 of 43 (58%) centers in 14 of 17 (82%) countries. The aSH population included 45 and 37 patients at weeks 52 and 104, respectively, and the Poznanski population included 32 and 26 patients at weeks 52 and 104, respectively.

In total, 188 patients were enrolled in the pcJIA trial and received ≥ 1 dose of tocilizumab. Baseline and ≥ 1 postbaseline aSH scores and Poznanski scores were assessed for 87 and 61 patients, respectively, from 25 of 43 (58%) centers in 14 of 17 (82%) countries. In the pcJIA study, 45 patients in the aSH population and 35 patients in the Poznanski population received tocilizumab continuously (continuous TCZ population). The aSH population included 40 and 35 patients at weeks 52 and 104, respectively, and the Poznanski population included 31 and 25 patients at weeks 52 and 104, respectively**.**

For both studies, baseline demographics and disease characteristics of patients who underwent radiographic assessments were similar to those of the total populations [19, 20] (Table 1).

**Table 1:** Baseline demographics and disease characteristics of patients with sJIA and pcJIA (radiographic and study populations)

Characteristic	sJIA			pcJIA		
	Adapted SH, *n* = 47	Poznanski, *n* = 33	All, *n* = 112	Adapted SH, *n* = 45	Poznanski, *n* = 35	All, *n* = 188
Age, years	9.9 (4.3)	8.4 (4.2)	9.7 (4.6)	10.8 (3.7)	9.9 (3.3)	11.0 (4.0)
Female, *n* (%)	24 (51)	13 (39)	56 (50)	34 (76)	27 (77)	144 (77)
Race, white, *n* (%)	43 (91)	29 (88)	99 (88)	33 (73)	25 (71)	150 (80)
Body weight, kg	33.7 (15.7)	28.2 (14.5)	33.8 (19.6)	39.3 (16.3)	36.3 (14.7)	39.6 (17.3)
Disease duration, years	5.2 (4.2)	4.8 (4.1)	5.2 (4.1)	3.9 (3.3)	3.2 (2.4)	4.2 (3.7)
Joints with active arthritis^a^	21.3 (15.7)	19.2 (16.5)	19.8 (15.7)	20.9 (13.7)	21.7 (14.5)	20.3 (14.3)
Joints with LOM^b^	20.0 (15.7)	18.2 (16.7)	19.8 (15.6)	14.8 (12.0)	16.3 (13.0)	17.6 (14.4)
Patient/parent global assessment VAS^c^	55.4 (22.7)	55.6 (25.1)	58.7 (24.4)	42.5 (26.3)	41.9 (26.4)	52.9 (25.0)
Physician global assessment VAS^c^	62.3 (19.5)	62.8 (20.0)	64.9 (22.3)	57.2 (19.8)	59.1 (18.4)	61.4 (20.7)
CHAQ-DI score (0–3)	1.6 (0.9)	1.6 (0.9)	1.7 (0.9)	1.3 (0.6)	1.3 (0.5)	1.4 (0.7)
ESR, mm/h	53.9 (31.5)	59.2 (35.2)	57.6 (34.2)	29.9 (22.3)	30.9 (21.9)	34.8 (25.5)
Previous DMARDs use, *n* (%)	34 (72)	22 (67)	82 (73)	31 (69)	20 (57)	134 (71)
Previous biologic use, *n* (%)	39 (83)	26 (79)	92 (82)	9 (20)	7 (20)	61 (32)
Background methotrexate use, *n* (%)	34 (72)	28 (85)	77 (69)	39 (87)	30 (86)	148 (79)
Methotrexate dose, mg/m^2^/week	–	–	–	12.5 (3.3)	12.7 (3.3)	13.0 (5.8)
Background oral GC use, *n* (%)	23 (49)	12 (36)	55 (49)	19 (42)	15 (43)	86 (46)
GC dose, mg/kg/day^d^	0.28 (0.17)	0.31 (0.16)	0.30 (0.20)	0.13 (0.05)	0.14 (0.05)	0.13 (0.05)

Data are mean (SD) unless otherwise noted

*Abbreviations*: *CHAQ-DI* Childhood Health Assessment Questionnaire–Disability Index, *DMARDs* disease-modifying antirheumatic drugs, *ESR* erythrocyte sedimentation rate, *GC* glucocorticoid, *LOM* limitation of motion, *pcJIA* polyarticular-course juvenile idiopathic arthritis, *SD* standard deviation, *SH* Sharp–van der Heijde, *sJIA* systemic juvenile idiopathic arthritis, *VAS* visual analog scale

^a^71-joint count

^b^67-joint count

^c^0–100 mm

^d^Prednisone equivalent

Notably, the Poznanski score could not be assessed in patients with advanced carpometacarpal erosions that made it difficult to define the bone ends or in older children who had apparent radiographic closure of the second metacarpal growth plate. These phenomena did not preclude assessment of the aSH score, which explains why patients with available aSH scores outnumber those who had the Poznanski score calculated.

### Inter-reader and intra-reader reliability

**Fig. 1:**
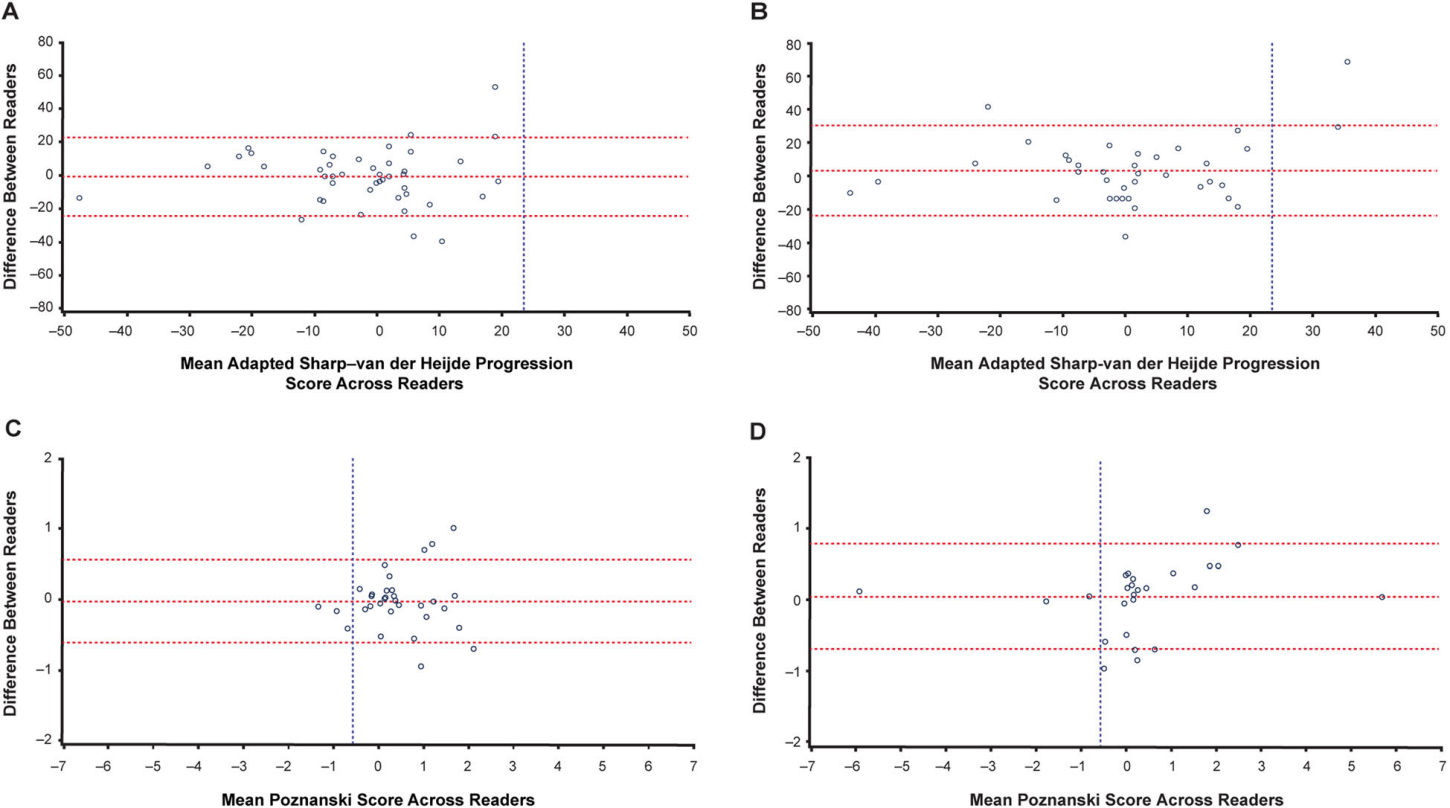
Bland and Altman plots of radiographic progression based on SDD in patients with sJIA. **a**, **b** Mean adapted SH progression scores across two reviewers for each patient with radiographic scores at baseline and week 52 (**a**, *n* = 45) and baseline and week 104 (**b**, *n* = 37). **c**, **d** Mean Poznanski score across two reviewers for each patient with Poznanski scores at baseline and week 52 (**c**, *n* = 32) and baseline and week 104 (**d**, *n* = 26). SDD thresholds are represented by vertical dashed lines. Patients with adapted SH progression are represented to the right of the vertical line in **a** and **b**. Patients with Poznanski progression are represented to the left of the vertical line in **c** and **d**. Horizontal dashed lines represent mean difference between readers ± SDD. In case of discrepancy between readers, radiographs were adjudicated and reread independently so consensus could be reached. SDD, smallest detectable difference; SH, Sharp–van der Heijde; sJIA, systemic juvenile idiopathic arthritis

**Fig. 2:**
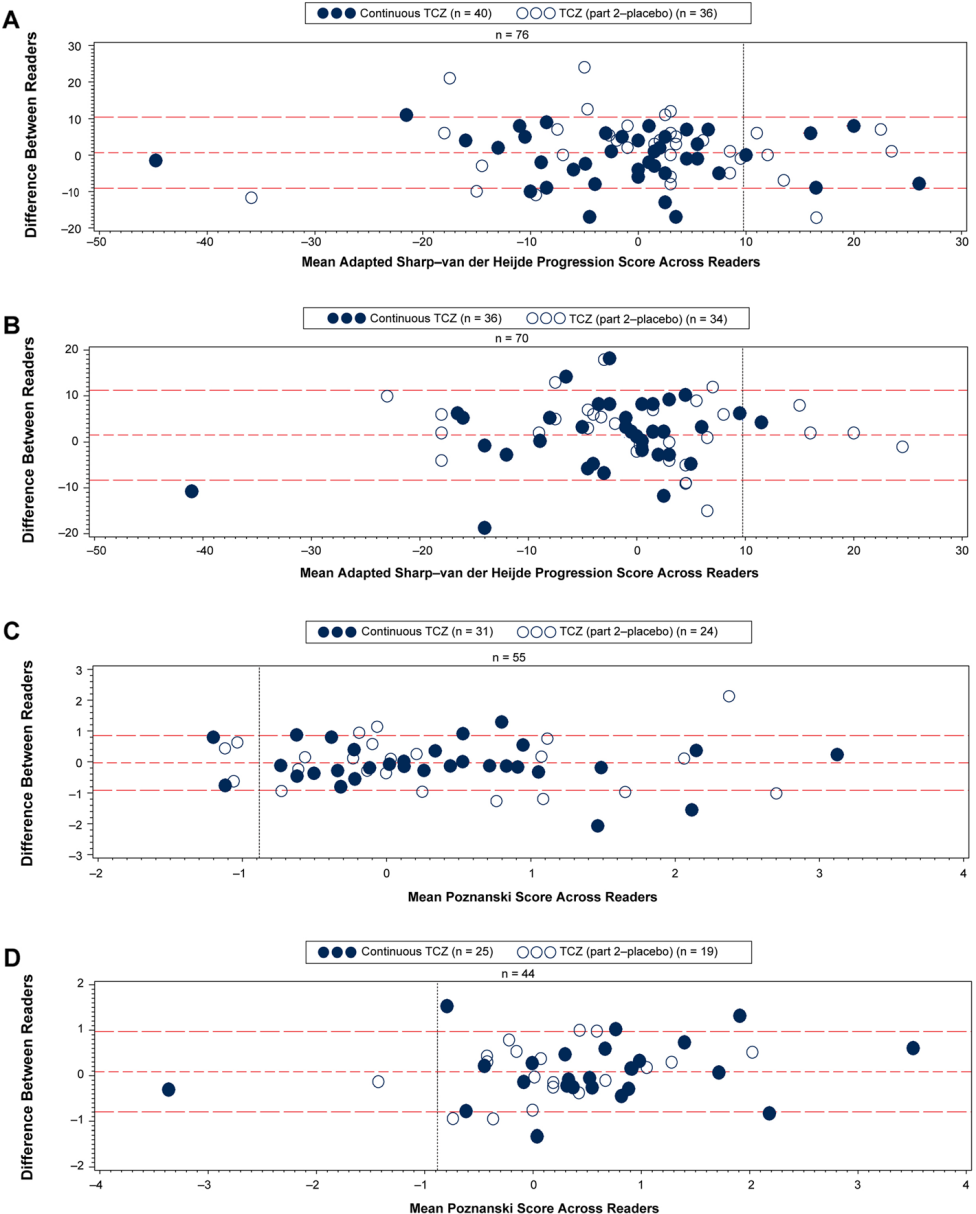
Bland and Altman plots of radiographic progression based on SDD in patients with pcJIA. **a**, **b** Mean adapted SH progression scores across two reviewers for each patient with radiographic scores at baseline and week 52 (**a**, *n* = 76) and baseline and week 104 (**b**, *n* = 70). **c**, **d** Mean Poznanski scores across two reviewers for each patient with Poznanski scores at baseline and week 52 (**c**, *n* = 55) and baseline and week 104 (**d**, *n* = 44). SDD thresholds are represented by vertical dashed lines. Patients with adapted SH progression are represented to the right of the vertical line in **a** and **b**. Patients with Poznanski progression are represented to the left of the vertical line in **c** and **d**. Patients randomly assigned to tocilizumab in part 2 are represented by filled circles; those randomly assigned to placebo in part 2 are represented by empty circles. Horizontal dashed lines represent mean differences between readers ± SDD. In case of discrepancy between readers, radiographs were adjudicated and reread independently so consensus could be reached. pcJIA, polyarticular-course juvenile idiopathic arthritis; SDD, smallest detectable difference; SH, Sharp–van der Heijde; TCZ, tocilizumab

Inter-reader and intra-reader agreement, as assessed by intra-class correlation coefficient (ICC) for radiographic scores, was good for both JIA subtypes and for both scoring methods, with all ICCs > 0.8 and most > 0.9 (results not shown). Agreement between readers was confirmed by Bland and Altman plots (Figs. 1 and 2).

### Radiographic progression in patients with sJIA

**Fig. 3:**
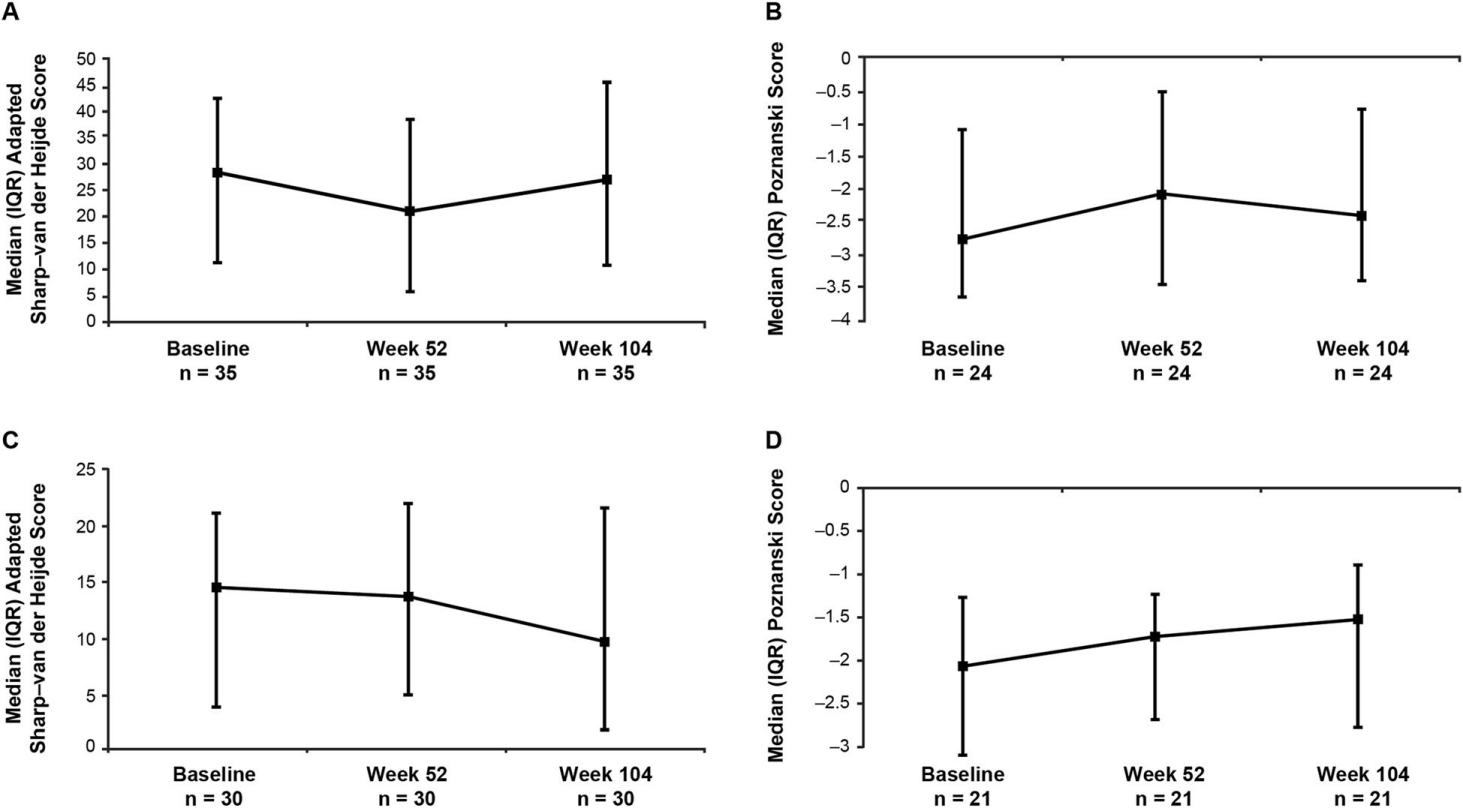
Radiographic scores at baseline, week 52, and week 104 for patients with sJIA and pcJIA. **a** Adapted SH scores and **b** Poznanski scores for patients with sJIA. **c** Adapted SH scores and **d** Poznanski scores for patients with pcJIA. SH score: higher score indicates greater damage. Poznanski score: the more negative a Poznanski score, the more severe the radiographic damage. IQR, interquartile range; pcJIA, polyarticular-course juvenile idiopathic arthritis; SH, Sharp–van der Heijde; sJIA, systemic juvenile idiopathic arthritis

Median [interquartile range] changes from baseline in total aSH scores were not significant at weeks 52 (0.00 [− 8.70, 4.00]); *P* = 0.302) and 104 (0.50 [− 7.50, 12.00]; *P* = 0.695) (Fig. 3a, Table 2). Similarly, no significant change from baseline was observed in erosion scores at weeks 52 (0.50 [− 3.50, 1.50]; *P* = 0.677) and 104 (0.50 [− 1.00, 4.50]; *P* = 0.257) or in JSN scores at weeks 52 (0.00 [− 4.00, 3.00]; *P* = 0.257) and 104 (0.00 [− 5.50, 4.00]; *P* = 0.937) (Table 2). Median [interquartile range] Poznanski scores increased significantly from baseline to week 52 (0.29 [− 0.05, 1.05]; *P* = 0.003), but change from baseline to week 104 was not significant (0.16 [− 0.01, 1.04]; *P* = 0.057) (Fig. 3b, Table 2). There was a weak negative correlation between aSH and Poznanski scores at week 52 (Pearson correlation, − 0.233; Spearman correlation, − 0.121) and a weak-to-moderate negative correlation at week 104 (Pearson correlation, − 0.682; Spearman correlation, − 0.303).

**Table 2:** Change from baseline in adapted SH and Poznanski scores

Score	Baseline		Change from baseline to week 52			Change from baseline to week 104		
	*n*	Median (IQR)	*n*	Median (IQR)	*P*	*n*	Median (IQR)	*P*
sJIA								
Adapted total SH	47	24.60 (8.50, 39.50)	45	0.00 (− 8.70, 4.00)	0.302	37	0.50 (− 7.50, 12.00)	0.695
Erosion	47	8.50 (1.50, 23.50)	45	0.50 (− 3.50, 1.50)	0.677	37	0.50 (− 1.00, 4.50)	0.257
JSN	47	13.00 (6.00, 19.50)	45	0.00 (− 4.00, 3.00)	0.257	37	0.00 (− 5.50, 4.00)	0.937
Poznanski	33	− 2.38 (− 3.48, − 0.84)	32	0.29 (− 0.05, 1.05)	0.003	26	0.16 (− 0.01, 1.04)	0.057
pcJIA								
Adapted total SH	45	8.00 (3.00, 18.50)	40	0.50 (− 7.25, 4.50)	0.700	35	− 1.00 (− 6.50, 2.50)	0.109
Erosion	45	3.00 (0.00, 6.50)	40	0.00 (− 1.50, 1.50)	0.819	36	0.00 (− 2.75, 0.75)	0.402
JSN	45	5.00 (1.00, 14.50)	40	0.25 (− 5.25, 3.50)	0.614	35	− 1.00 (− 3.50, 1.50)	0.109
Poznanski	35	− 1.45 (− 2.51, − 0.75)	31	0.26 (− 0.34, 0.91)	0.077	25	0.55 (0.04, 0.92)	0.004

*Abbreviations: IQR* interquartile range, *JSN* joint space narrowing, *pcJIA* polyarticular-course juvenile idiopathic arthritis, *SH* Sharp–van der Heijde, *sJIA* systemic juvenile idiopathic arthritis

SH score: higher score indicates greater damage. Poznanski score: the more negative a Poznanski score, the more severe the radiographic damage

The SDD for aSH score progression was 23.6 at week 52 and 27.4 at week 104. Using the SDD of 23.6, 100% and 94.6% of tocilizumab-treated patients showed no aSH progression at weeks 52 and 104, respectively (Fig. 1a, b). Proportions of patients without progression—using a cutoff of zero—are described in the Additional File.

The SDD for Poznanski score progression was 0.58 at week 52 and 0.74 at week 104. Based on the SDD of 0.58, 90.6% and 88.5% of patients did not experience Poznanski progression at weeks 52 and 104, respectively (Fig. 1c, d).

Mean annualized progression rates from baseline to week 104 were 0.29, 0.44, and − 0.15 for total, erosion, and JSN aSH scores, respectively, and − 0.18 for Poznanski score, indicating a lack of radiographic progression over 2 years.

The clinical effectiveness of tocilizumab, assessed by JIA American College of Rheumatology (ACR) response criteria [29], did not appear to be related to radiographic progression. All patients with available radiographs achieved JIA ACR50 response or higher, except for one who was a nonresponder at week 52 but who achieved JIA ACR70 response by week 104; this patient did not experience aSH or Poznanski progression according to SDD. Two patients who exhibited aSH progression at week 104 (based on SDD calculation) met JIA ACR90 criteria. One of these patients also exhibited Poznanski progression. Of the other two patients with Poznanski progression at week 104, one achieved JIA ACR70 and the other achieved JIA ACR90 response.

### Radiographic progression in patients with pcJIA

There were nonsignificant changes in total, erosion, and JSN aSH scores from baseline to weeks 52 and 104 (Fig. 3c, Table 2). Patients receiving glucocorticoids at baseline had a positive median change in aSH score, whereas those not receiving glucocorticoids at baseline had a negative median change (2.50 vs − 1.50). No significant differences were observed for other parameters (data not shown). A significant positive change of 0.55 (*P* = 0.004) in Poznanski score was observed from baseline to week 104, and a positive, though not significant, change of 0.26 (*P* = 0.077) was detected from baseline to week 52 (Fig. 3d, Table 2). There was a weak-to-moderate negative correlation between aSH and Poznanski scores at week 52 (Pearson correlation, − 0.420; Spearman correlation, − 0.450) and week 104 (Pearson correlation, − 0.522; Spearman correlation, − 0.429).

Based on week 104 data, SDD values were 9.76 and − 0.88 for aSH and Poznanski progression, respectively. Using these SDDs, 87.5% and 97.1% of patients in the continuous TCZ population experienced no aSH progression at weeks 52 and 104, respectively, and one patient experienced progression after 2 years of tocilizumab treatment (Fig. 2a, b). Using the same SDD, 92.9% of patients in the all TCZ population experienced no aSH progression at week 104 (Fig. 2b).

Using the SDD of − 0.88, 96.0% of patients in the continuous TCZ population experienced no Poznanski progression at week 104 compared with 93.5% at week 52 (Fig. 2c, d). Applying the same SDD, 95.5% of patients in the all TCZ population experienced no Poznanski progression at week 104 (Fig. 2d).

**Fig. 4:**
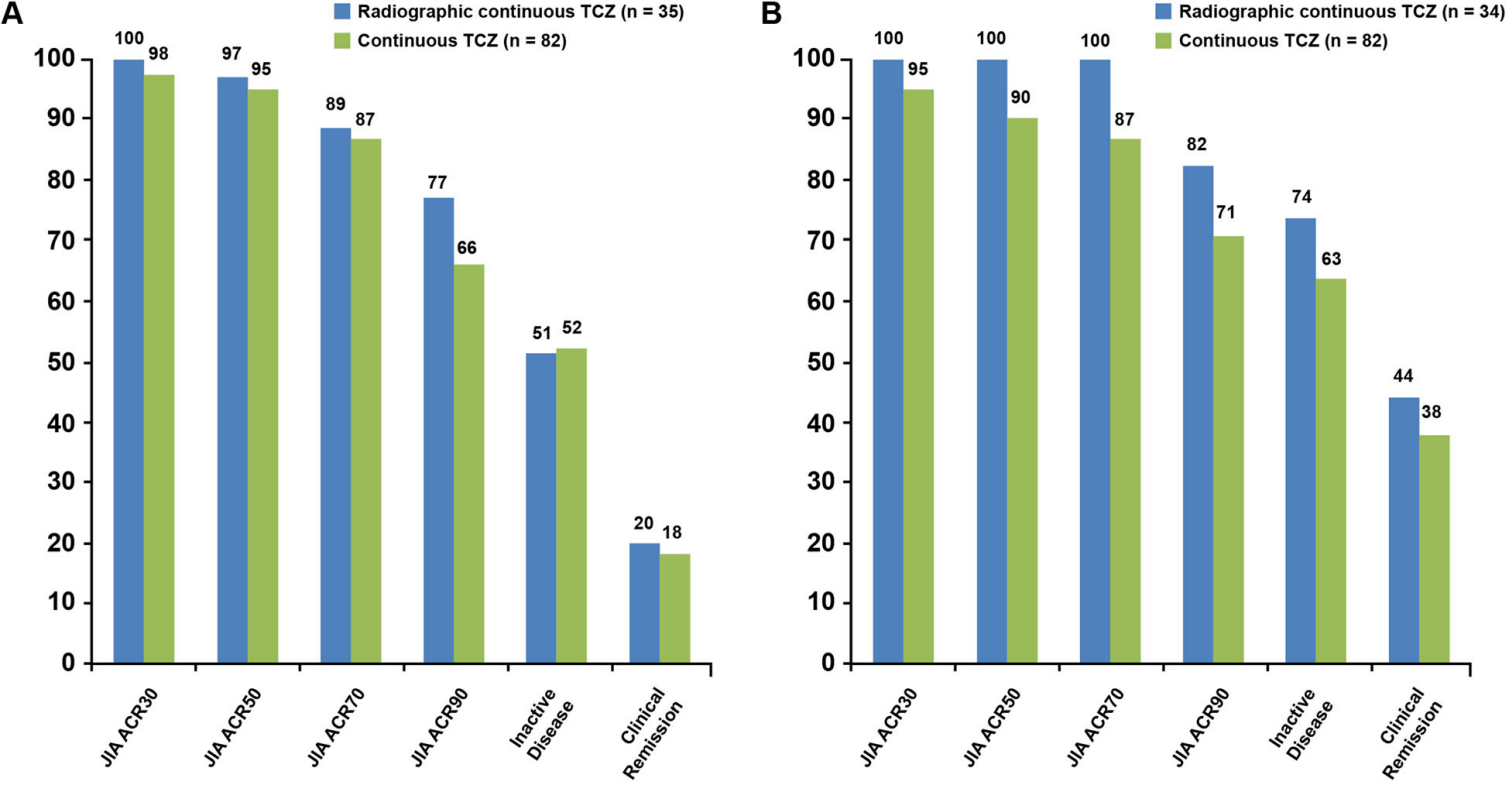
Efficacy for patients who did not experience radiographic progression in the pcJIA trial. **a** Efficacy response for patients who did not experience adapted SH progression based on SDD at week 52 compared with response rates for all patients in the continuous TCZ population. **b** Efficacy response for patients who did not experience adapted SH progression based on SDD at week 104 compared with response rates for all patients in the continuous TCZ population. JIA ACR, juvenile idiopathic arthritis American College of Rheumatology response; pcJIA, polyarticular-course juvenile idiopathic arthritis; SDD, smallest detectable difference; SH, Sharp–van der Heijde; TCZ, tocilizumab

Mean annualized progression rates from baseline to week 104 were calculated in the all TCZ population. Consistent with the continuous TCZ population, annualized progression rates for total, erosion, and JSN aSH scores were − 0.75, 0.31, and − 0.95, respectively, and the Poznanski score was 0.19, indicating a lack of radiographic progression in the entire radiographic population over 2 years. The JIA ACR response rate was higher in patients without radiographic progression than in the entire study population at weeks 52 and 104 (Fig. 4a, b). The proportion of patients in each JIA ACR response category was maintained or increased from week 52 to week 104 and was greater at week 104 than the proportions in the continuous TCZ population, which included all patients randomly assigned to tocilizumab during part 2, regardless of the availability of radiographic data (Fig. 4a, b).

## Discussion

TENDER and CHERISH are the first trials in JIA to include radiologic analysis. Most patients with sJIA and pcJIA treated with tocilizumab experienced no radiographic progression assessed by SDD and *zero value* methods. There was little change in radiographic scores of structural joint damage during 2 years of treatment. This is the first time that two separate phase 3 randomized controlled trials in patients with sJIA or pcJIA have been evaluated for X-ray progression within the framework of a pediatric investigation plan for the European Medicines Agency and a pediatric study plan for the US Food and Drug Administration.

The present analysis was not controlled; therefore, the possibility that patients had less aggressive illness than those who were not included in the analysis cannot be excluded. However, all JIA outcome measures at the start of treatment were comparable between patients included and excluded from the analysis. Furthermore, all patients had severe, long-standing systemic or polyarticular-course disease that was not controlled despite previous use of at least one traditional second-line agent, and all had bilateral wrist involvement. Patients with sJIA and pcJIA are more likely to develop destructive disease [30–32], and it has been suggested that patients with JIA and bilateral wrist disease are at high risk for radiographic progression [14, 33].

The observed changes in aSH score in pcJIA patients and the change in Poznanski score in sJIA and pcJIA patients suggest that at least some patients experienced improvement of articular damage. Altogether, these findings indicate that tocilizumab is potentially capable of halting the progression of radiographic joint damage in children with JIA. Improvement in the rate of radiographic progression in children with JIA is not surprising because the regenerative capacity of articular cartilage is better in growing children than in adults [4].

These observations corroborate previous reports showing amelioration of radiographic joint changes with tocilizumab treatment in children with systemic JIA [16, 25]. Coupled with previous demonstrations of the potential capacity of etanercept to repair radiographic joint damage in patients with JIA [15, 34], these observations indicate that biologic agents may have disease-modifying potential in JIA and underscore the need for randomized controlled trials to explore the capacity of biologics to prevent structural joint damage.

Given the high degree of concomitant methotrexate use among patients in the sJIA and the pcJIA studies, our data do not establish whether combination with methotrexate could enhance the effectiveness of tocilizumab. Literature is inconclusive on the effects of methotrexate on joint destruction, although two studies have suggested that this medication might have disease-modifying potential [11, 13, 14, 35]. Notably, all previous investigations of the effect of disease-modifying antirheumatic drugs on radiographic progression in JIA in wrist joints used the Poznanski score.

We used aSH and Poznanski scores to measure radiographic progression in the present analysis because these methods were specifically developed and validated for use in patients with JIA [4, 6, 15]. These measures significantly correlated with long-term joint damage and disability in physical function of children with pcJIA [6]. In the present analysis, radiography scores were reliable and showed good inter-reader and intra-reader agreement. In the absence of radiographic progression, approximately 50% of patients are expected to experience change from baseline of less than zero, and approximately 50% are expected to experience change greater than zero because of random errors introduced during radiographic reading. Therefore, the approximate 50:50 split between patients with and without radiographic progression at weeks 52 and 104 suggests there was little radiographic progression throughout the study, which is consistent with small changes in aSH score.

Limitations of the present analysis include the small number of patients in the radiographic populations of both trials. Limited availability of radiographs in the patient sample was due to nonparticipation of some study investigators in the radiographic study or lack of consent for radiographic assessments. Both scoring systems used to quantify radiographic damage evaluated wrist and hand joints, and it is unclear whether changes in these joints sufficiently reflect damage to large weight-bearing joints [4]. Some radiographs could not be assessed with the Poznanski method because of advanced bone erosions, which precluded a reliable definition of bone ends, or because of radiographic closure of growth plates of the second metacarpal bone in postpubertal patients. Lastly, we recognize that it was not possible to compare radiographic progression of tocilizumab-treated patients with that of patients who did not receive tocilizumab, particularly in the context of published data regarding the rate of radiographic progression in sJIA and pcJIA patients who did not receive tocilizumab.

## Conclusion

In conclusion, this analysis of radiographic data from the TENDER and CHERISH trials suggests that tocilizumab may prevent radiographic progression in children with sJIA and children with pcJIA. However, because the natural course of arthritis in children is heterogeneous and a control group not exposed to tocilizumab was not available, we cannot draw definitive conclusions regarding the ability of tocilizumab to halt or diminish radiographic progression in JIA, and our findings should be confirmed in future studies.

## Supplementary information

**Additional file 1 Supplemental Methods**. Inter-reader reliability methods for assessment of radiographs. Radiographic progression using cutoff of zero.

## Data Availability

Qualified researchers may request access to data through the clinical study data request platform (www.clinicalstudydatarequest.com). Further details on Roche’s criteria for eligible studies are available here: https://clinicalstudydatarequest.com/Study-Sponsors/Study-Sponsors-Roche.aspx. For further details on Roche’s Global Policy on the Sharing of Clinical Information and how to request access to related clinical study documents, see here: https://www.roche.com/research_and_development/who_we_are_how_we_work/clinical_trials/our_commitment_to_data_sharing.htm.

## Abbreviations

ACR: American College of Rheumatology
aSH: Adapted Sharp–van der Heijde
CHAQ-DI: Childhood Health Assessment Questionnaire–Disability Index
DMARDs: Disease-modifying antirheumatic drugs
ESR: Erythrocyte sedimentation rate
GC: Glucocorticoid
ICC: Intra-class correlation coefficient
IL-6: Interleukin-6
IQR: Interquartile range
JIA ACR: Juvenile idiopathic arthritis American College of Rheumatology response
JSN: Joint space narrowing
LOM: Limitation of motion
M2: Second metacarpal bone
pcJIA: Polyarticular-course juvenile idiopathic arthritis
PRCSG: Pediatric Rheumatology Collaborative Study Group
PRINTO: Paediatric Rheumatology International Trials Organisation
RM: Radial metacarpal
SD: Standard deviation
SDD: Smallest detectable difference
SH: Sharp–van der Heijde
sJIA: Systemic juvenile idiopathic arthritis
TCZ: Tocilizumab
VAS: Visual analog scale
