# Supplementary material for: Tocilizumab may slow radiographic progression in patients with systemic or polyarticular-course juvenile idiopathic arthritis: post hoc radiographic analysis from two randomized controlled trials
Source: Arthritis Res Ther. 2020 Sep 10;22:211. doi: 10.1186/s13075-020-02303-y (PMC7488325; doi:10.1186/s13075-020-02303-y)
Supplement: Supplementary file 1 — Additional file 1 Supplemental Methods. Inter-reader reliability methods for assessment of radiographs. Radiographic progression using cutoff of zero. [file 13075_2020_2303_MOESM1_ESM.docx]

**ADDITIONAL FILE 1**

**Inter-reader reliability methods for assessment of radiographs**

Calibration of readers was performed by independent reading of 10 radiographs, and disagreement was resolved by consensus. To assess inter-reader reliability, another set of 15 radiographs was read twice by each reader 4 weeks apart. Recalibration was performed if the intraclass correlation coefficient (ICC) was ≤0.8. Radiographs used for calibration were not included in subsequent reading procedures. Each reader was assigned the Poznanski score first and the adapted Sharp–van der Heijde (aSH) score second in a set of 30 radiographs. Reading of the remaining radiographs continued if the interreader ICC was >0.8; otherwise, a retraining session was performed. Intra-reader reliability was assessed using 30 random radiographs >28 days after the previous reading (ICC >0.8 was required). Outliers of discordant evaluations between readers were identified using Bland and Altman plots, and discrepancies were resolved by consensus.

**Additional details for calculation of aSH score**

In younger children (bone age <5 years for boys and <6 years for girls), some wrist areas were not assessable because of incomplete ossification of the carpal bones. If complete scoring of X-ray indices was not possible, summary scores were derived from the average score of interpretable areas, rounded to the closest integer.

The degree of ossification and the width of joint spaces vary with age in childhood; therefore, evaluation of JSN and bony erosion in an individual patient over time is difficult, as is comparison of films from patients of different ages. To facilitate assessment of JSN and bone erosion, patient radiographs were compared with radiographs from sex-matched healthy children of the same skeletal age and evaluated according to the Greulich-Pyle atlas [1].

**Additional details for calculation of Poznanski score**

Patients with advanced carpometacarpal erosions that made it difficult to define the bone ends and patients with apparent radiographic closure of the second metacarpal growth plate were excluded from the analysis. Measurements were made using an electronic ruler accurate to one decimal point. The worst left and right Poznanski scores for each patient were used in the analyses.

**Assessment of radiographic progression**

Radiographic progression was determined by subtracting baseline scores from week 52 and 104 scores. Radiographic progression for aSH and Poznanski scores was defined using the smallest detectable difference (SDD) and the zero value [2].

Inter-reader differences in change from baseline values were determined, and SDD was then calculated using the following formula:

SDD = t_0.05_, _n – 1_ × (SD_diff_/$\surd$2)

SD_diff_ is the standard deviation of difference between readers in change from baseline values. If the SDD differed between weeks 52 and 104, the more conservative (lower) value was used as the cutoff for both time points.

A positive change in aSH score [1] or a negative change in Poznanski score [3], or both, was considered indicative of radiographic progression. Therefore, absence of aSH score progression was defined as a change from baseline in aSH score of ≤SDD or zero, whereas absence of Poznanski score progression was defined as a change from baseline in Poznanski score of ≥–SDD or zero. Data are reported using Bland and Altman plots.

**Radiographic progression using cutoff of zero**

Using the cutoff of zero, the proportion of patients with sJIA who did not experience aSH progression decreased from 55.5% at week 52 to 48.6% at week 104. The proportion of patients who did not experience Poznanski progression was 75.0% at week 52 and 76.9% at week 104.

When zero was used as a cutoff value for pcJIA patients, the proportion of patients in the Continuous TCZ population who did not experience aSH progression increased from week 52 (50.0%) to week 104 (57.1%). Based on the zero cutoff, 61.3% and 76.0% of patients in the Continuous TCZ population did not experience Poznanski progression at weeks 52 and 104, respectively.

**References**

1. Ravelli A, Ioseliani M, Norambuena X, [Sato J](https://www.ncbi.nlm.nih.gov/pubmed/?term=Sato%20J%5BAuthor%5D&cauthor=true&cauthor_uid=17763418), [Pistorio A](https://www.ncbi.nlm.nih.gov/pubmed/?term=Pistorio%20A%5BAuthor%5D&cauthor=true&cauthor_uid=17763418), [Rossi F](https://www.ncbi.nlm.nih.gov/pubmed/?term=Rossi%20F%5BAuthor%5D&cauthor=true&cauthor_uid=17763418), et al. Adapted versions of the Sharp/van der Heijde score are reliable and valid for assessment of radiographic progression in juvenile idiopathic arthritis. Arthritis Rheum. 2007;56(9):3087–95.

2. Lassere M, Boers M, van der Heijde D, [Boonen A](https://www.ncbi.nlm.nih.gov/pubmed/?term=Boonen%20A%5BAuthor%5D&cauthor=true&cauthor_uid=10090192), [Edmonds J](https://www.ncbi.nlm.nih.gov/pubmed/?term=Edmonds%20J%5BAuthor%5D&cauthor=true&cauthor_uid=10090192), [Saudan A](https://www.ncbi.nlm.nih.gov/pubmed/?term=Saudan%20A%5BAuthor%5D&cauthor=true&cauthor_uid=10090192), et al. Smallest detectable difference in radiological progression. J Rheumatol. 1999;26(3):731–9.

3. Poznanski AK, Hernandez RJ, Guire KE, [Bereza UL](https://www.ncbi.nlm.nih.gov/pubmed/?term=Bereza%20UL%5BAuthor%5D&cauthor=true&cauthor_uid=725041), [Garn SM](https://www.ncbi.nlm.nih.gov/pubmed/?term=Garn%20SM%5BAuthor%5D&cauthor=true&cauthor_uid=725041). Carpal length in children—a useful measurement in the diagnosis of rheumatoid arthritis and some congenital malformation syndromes. Radiology. 1978;129(3):661–8.
